# A Weakly Supervised Deep Learning Framework for Sorghum Head Detection and Counting

**DOI:** 10.34133/2019/1525874

**Published:** 2019-06-27

**Authors:** Sambuddha Ghosal, Bangyou Zheng, Scott C. Chapman, Andries B. Potgieter, David R. Jordan, Xuemin Wang, Asheesh K. Singh, Arti Singh, Masayuki Hirafuji, Seishi Ninomiya, Baskar Ganapathysubramanian, Soumik Sarkar, Wei Guo

**Affiliations:** ^1^Department of Mechanical Engineering, Iowa State University, Ames, IA, USA; ^2^Department of Computer Science, Iowa State University, Ames, IA, USA; ^3^CSIRO Agriculture and Food, St. Lucia, QLD, Australia; ^4^School of Agriculture and Food Sciences, The University of Queensland, Gatton, QLD 4343, Australia; ^5^Queensland Alliance for Agriculture and Food Innovation (QAAFI), The University of Queensland, Gatton, QLD, Australia; ^6^Queensland Alliance for Agriculture and Food Innovation (QAAFI), The University of Queensland, Warwick, QLD, Australia; ^7^Department of Agronomy, Iowa State University, Ames, IA, USA; ^8^International Field Phenomics Research Laboratory, Institute for Sustainable Agro-Ecosystem Services, Graduate School of Agricultural and Life Sciences, The University of Tokyo, Tokyo, Japan

## Abstract

The yield of cereal crops such as sorghum (*Sorghum bicolor* L. Moench) depends on the distribution of crop-heads in varying branching arrangements. Therefore, counting the head number per unit area is critical for plant breeders to correlate with the genotypic variation in a specific breeding field. However, measuring such phenotypic traits manually is an extremely labor-intensive process and suffers from low efficiency and human errors. Moreover, the process is almost infeasible for large-scale breeding plantations or experiments. Machine learning-based approaches like deep convolutional neural network (CNN) based object detectors are promising tools for efficient object detection and counting. However, a significant limitation of such deep learning-based approaches is that they typically require a massive amount of hand-labeled images for training, which is still a tedious process. Here, we propose an active learning inspired weakly supervised deep learning framework for sorghum head detection and counting from UAV-based images. We demonstrate that it is possible to significantly reduce human labeling effort without compromising final model performance (*R*^2^ between human count and machine count is 0.88) by using a semitrained CNN model (i.e., trained with limited labeled data) to perform synthetic annotation. In addition, we also visualize key features that the network learns. This improves trustworthiness by enabling users to better understand and trust the decisions that the trained deep learning model makes.

## 1. Introduction

Sorghum (*Sorghum bicolor* L. Moench) is a C4 tropical grass that plays an essential role in providing nutrition to humans and livestock, particularly in marginal rainfall environments. The timing of head development and the number of heads per unit area are key adaptation traits to consider in breeding programs, and this requires breeders to investigate and record the appearance of heads in the field plot by plot. However, for crops like sorghum, each planted seed can give rise to multiple tillers. Consequently, the number of heads per unit area can be greater than the number of plants, which dramatically increases the difficulties for such a survey, especially for large-scale breeding trails (namely, 100+ heads per plot times 1000+ plots, which is 100000+ when it comes to counting). So, in most cases, breeders will often investigate a part of the plot [1] (put a 1-meter stick randomly inside a plot and count the head number around it) or even skip this step to only measure grain size or weight from subsampled heads during harvest. Those alternative approaches fail to fairly represent variations within the entire plot, especially for breeding populations. This is especially important when it comes to providing information on the genotypic variation in the number of fertile heads produced per unit.

Machine learning and image processing have proved their utility in diverse fields. Especially in the field of plant phenotyping [2–7], these tools have laid a strong foundation in detecting multiple crop diseases [8] as well as making sense of disease severity without the need for any additional human supervision [8], crop/weed discrimination [9–12], canopy/individual extraction [13, 14], fruit counting/flowering [15–17], and head/ear/panicle counting [18–20]. Our hypothesis is that machine learning and image processing along with unmanned aerial vehicles (UAV) based photogrammetry is a reliable alternative to the labor-intensive sorghum head survey in the field [21–23]. To the best of our knowledge, only one study has reported an approach to detect and count sorghum head from UAV images in recent literature [24]. The study uses carefully selected color and morphology features to train a two-step machine learning model to detect and count the number of heads from UAV images. It produces a good counting accuracy at the field level, but faces difficulties when it comes to the segmented single plot images because parts of the head along the plot boundaries have been cut out which leads to a loss in their morphological features.

Recently, performance of deep learning-based object detection has improved dramatically and has especially been useful for detecting partially occluded objects. However, as in case of most deep learning-based algorithms, deep learning models for object detection task require a massive amount of labeled images for training, in order to reach a desired level of accuracy. This, again, is labor-intensive and tedious. In order to overcome this limitation, researchers have proposed data augmentation (e.g., mirror-reverse, rotation, geometric transformation, scaling/zooming, contrast random transformation, random noise generation, and random erasing of the original image) approaches to increase the number of training images [25]. However, in many cases, generic data augmentation fails and smart choices have to be made to augment the dataset for properly training the network [26].

In this paper, we leverage another concept that is known as the* weakly supervised learning* to significantly reduce the cost of human labeling [27, 28]. There are several variants of weak supervision reported in literature. One such variant involves incomplete supervision [29] where a small section of the training data is labeled and the remaining large section of data is unlabeled. In this situation, a machine learning (ML) model is usually trained partially with the labeled part of the data and then that semitrained model is used on the unlabeled section of data to improve the model. There are again several possible strategies for such improvement. A simple strategy is known as self-training or bootstrapping [30] that involves running inference with the semitrained model for labeling the unlabeled data which can then be used for retraining the model. Note that such labeling could have a lot of inaccuracies, also known as noisy labels. Therefore, in order to function as intended, the initial semitrained model needs to be sufficiently accurate. As this is a rather stringent requirement in practice, ML practitioners often involve an oracle (e.g., human annotator) to verify the annotations performed by the semitrained model. However, rather than asking the oracle to verify/provide annotations for all unlabeled data, typically a much smaller number of* optimally* selected data samples are chosen for human labeling/verification. This class of weak supervision techniques is known as active learning [31, 32], which has been widely studied in the context of shallow [33, 34] and deep machine learning [35, 36]. While this is the primary motivation of our proposed framework, we do not select such an optimal subset of data samples for human verification. Instead, we select a random subset of data samples and present their labels (generated by the semitrained model) for human verification. Therefore, we simply refer to our proposed scheme as a weakly supervised deep learning framework. Note that one can also avoid involvement of human annotators altogether via employing semisupervised techniques [37, 38].

We compare the outcomes of the proposed framework with both human annotation of the images as well as human-counting of sorghum heads in the field. Note that, due to the size of the experiment-field, field counting by human counters was conducted with a subsampling strategy, that is, the current standard in breeding (This strategy involves placing a 1-meter stick randomly within a plot and counting the sorghum heads around it. This is done by breeders in the field for each plot.).

The main body of the paper other than Section 1 (this section) is organized as follows: Section 2 provides details on the experiments conducted in the field, data collection procedure, and preparing the data for further analysis.  Section 3 deals with the methodology we propose in this work and how it builds upon existing methods. Results are shown and elaborated upon in Section 4 and, lastly, Section 5 discusses the presented results and proposes future directions.

## 2. Field Experiment, Data Collection, and Preparation

The field experiment was conducted at Hermitage, Queensland, Australia (28.21°S, 152.10°E, 459 m above sea level) during the 2015-16 summer growing season and the seeds were sowed on 22nd December, 2015. The detailed information for field configuration and UAV flight design is described in our previous work [24]. From 2109 originally collected UAV images (resolution 5472 × 3648), 3D point cloud and orthomosaic images were generated, which were then used to extract the individual plots using our previously reported method [24, 39]. Average plant density was 115,000 plants per hectare. Each plot consisted of two 5 m long rows. The trial used a solid row configuration with a row spacing of 0.76 m while the distance between two neighboring plots was 1 m. In total, 28825 individual plot images were extracted from original images with specific rotations applied for generating varying field orientations. There were a total of 1440 plots laid out as 36 columns × 40 rows with several columns (216 plots in total) being filler plots for spraying access. These 1440 images are a subset of the initially extracted 28825 images. Out of those 1440 plot images, 1269 were labeled.

We also use two separate datasets (“40Plots” and “52Croppped”) from our previous work [24] to compare the performance of the proposed method with the state-of-the-art results, reported there.

A web-based interactive labeling tool (see Figure S1) (Web Tool for Annotation) was designed to label the rectangular regions of interest (ROIs) of ground-truth data from images. The tool allows single or multiple human annotators to label the same set of image data at the same time. Furthermore, all labeled data are subsequently validated by an administrator (who also serves as an expert in the field) to ensure the quality. The tool then exports the labeled data with a KITTI format text file. The validation protocol works as follows: first, a labeler works on the dataset and draws the bounding box around heads for labeling. The labels generated by the labeler will then be validated by an administrator. The administrator performs a quality control by checking to see if all labeled bounding boxes are correct. If they are correct, the labels are approved and saved to the database. If not, necessary modifications are done by the administrator or the image is sent back to the labeler for relabeling.

## 3. Methods

### 3.1. Deep Learning Framework

**Figure 1:**
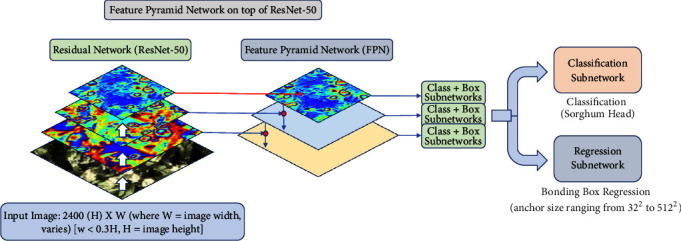
RetinaNet: the deep learning architecture adopted in our framework.

We devise a RetinaNet [40] based approach with the residual network, ResNet-50 [41] for our deep learning architecture as illustrated in Figure 1. Focal Loss [40] has been used as the loss function of choice for training the sorghum head detection network.

RetinaNet and Focal Loss have been well described in [40], but we discuss the same here briefly for the sake of completeness. RetinaNet is devised as a network consisting of a “backbone” network and two subnetworks (subnets) with specific functionalities. The former computes a convolutional feature map (F.M) for a given input image and, in our case, this “backbone” network is simply a convolutional (residual) network ResNet-50. After this feature map is computed, the first subnetwork performs convolutional object classification on the output that the residual network (ResNet-50) produces while the second subnet performs convolutional bounding box regression. The two subnets thus work in tandem and are specifically tailored and suitable for single-shot, dense detection problems. This unique capability of RetinaNet makes it especially suitable for our problem, considering we have image samples that have many target objects in each (both overlapping and nonoverlapping) for detection. We leverage this* dense* property of our dataset and use this specific method as a backbone for developing our framework.

#### 3.1.1. Backbone Network

The Feature Pyramid Network (FPN) [42] is adopted as the* backbone* network. FPNs are effective in constructing rich, multiscale feature pyramid (and generate several layered representations of features from target layers in the neural network) from an input image. From these pyramids that the FPN presents, every level of the pyramid can be queried for detecting objects at different scales. This improves multiscale predictions from fully convolutional networks. In our case, we build a FPN on top of the ResNet-50. We use pyramids with levels *P*_3_ through *P*_7_ (the default setting reported in [40]). A point to note here is that pyramid, *P*_*s*_, has resolution 2^*s*^ lower than the input, where *s* indicates pyramid level. All the pyramid levels have 256 channels. Details of the pyramid are in [42].

#### 3.1.2. Regression Subnetwork

For the regression subnet, anchor boxes similar to those in [42] were used. The lower limit on the area of the anchor boxes is 32^2^ and the upper limit is 512^2^ for pyramid levels *P*_3_ to *P*_7_, respectively. These anchor boxes are also translation invariant. Details of the anchor design are mentioned in [40, 42]. It is worth mentioning here that each anchor is assigned a length *K* one-hot vector of classification targets (for our case, *K* = 1 since we only have one class, i.e., Sorghum Head (SH)) and a 4-vector of box regression targets. We use an intersection-over-union (IoU) threshold of 0.5 to assign the anchors to ground-truth object boxes and reject assigning anchors if their IoU is in the range [0,0.5). We carry out a few experiments by varying the IoU threshold from 0.4 to 0.9 to see how the network performs in terms of mAP values. This is reported in Section 5. However, for most applications as well as for ours, an IoU threshold of 0.5 is acceptable.

#### 3.1.3. Classification and Prediction

To predict the probability of object presence at each spatial position for each of the anchors and the object class, we use a classification subnetwork (subnet). Since we have only one class (label), for prediction purposes, a simple residual network, ResNet-50, is sufficient. The classification subnet is attached to each Feature Pyramid Network (FPN) level with its parameters sharing across the different levels of the pyramid network. An input feature consisting of C (here, we have *C* = 256) channels from a target pyramid level is taken and four 3 × 3 convolutional (conv) layers each with C filters are applied, with ReLU activation on top. After that, another 3 × 3 conv layer with *A* (here, we have *A* = 9) filters is added. We then apply sigmoid activation to get a binary prediction per spatial location. Note that no parameters are shared between the box regression subnet (which we describe briefly in the next paragraph) and this particular object classification subnet.

#### 3.1.4. Bounding Box Regression

Parallel to the above-mentioned classification subnet, the box regression network is used to regress the offset from each anchor box to a nearby ground-truth object. A small-scale fully connected network is coupled with each pyramid level to carry out the regression task. The box regression subnet shares an identical design with the classification subnet with the only exception that it generates 4A linear outputs for every spatial location. Thus, for every A anchors for each spatial location, these four outputs help predict the relative offset between the ground-truth box and the anchors. The bounding box regressor used in this case is class-agnostic.

#### 3.1.5. Data Description and Loss Function

We initially collected 283 hand-labeled canopy images. Out of that initial pool of 283 images each constituting of 2 rows of sorghum plants, our training data includes a randomly selected subset of 40 images. This choice of 40 images is further discussed in Section 4. The network was initialized with random weights and the training was carried out for 40 epochs with a batch size of 10 to generate the model.

Focal Loss [40] extends the categorical cross entropy (CE) loss for binary classification as follows: first, CE loss is defined as

(1a) $$\begin{array}{c} CE\left(\alpha ,x\right)=-\log\left(\alpha \right)\text{if }x=1 \end{array}$$

(1b) $$\begin{array}{c} CE\left(\alpha ,x\right)=-\log\left(1-\alpha \right)otherwise \end{array}$$

where *x* is the ground-truth class and *α* ∈ [0,1] is the probability estimated by the model for class with label *x* = 1. To compress this CE loss definition, we further define

(2a) $$\begin{array}{c} \alpha_{t}=\alpha \text{if }x=1 \end{array}$$

(2b) $$\begin{array}{c} \alpha_{t}=1-\alpha otherwise \end{array}$$

Thus, *CE*(*α*, *x*) = *CE*(*α*_*t*_) = −log⁡(*α*_*t*_). Now, Focal Loss (FL) is defined as

(3) $$\begin{array}{c} FL\left(\alpha_{t}\right)=-{\left(1-\alpha_{t}\right)}^{\tau }\log\left(\alpha_{t}\right) \end{array}$$

where (1 − *α*_*t*_) is a modulating factor added to the cross entropy loss with *τ* ≥ 0 being a hyperparameter known as the focusing factor. Further, a balancing factor, *γ*, is added to the FL term to obtain slightly better accuracy:

(4) $$\begin{array}{c} FL\left(\alpha_{t}\right)=-\gamma_{t}{\left(1-\alpha_{t}\right)}^{\tau }\log\left(\alpha_{t}\right) \end{array}$$

Adam [43], with a learning rate of 0.00001 was used as the optimizer of choice.

#### 3.1.6. Training Hardware, Source Code, and Libraries Used

We train our network using a NVIDIA Tesla P40 GPU (22GB of GPU memory) while evaluation and inference were carried out using a NVIDIA GeForce GTX 1070 (8GB of GPU memory). Training, evaluation and testing were done using the Keras deep learning library with Tensorflow backend on Python 2.7. The source code on RetinaNet built for Keras was accessed from [44] and then modified for our problem. The codes used for generating results and reproducing the results presented in this work are available at DeepSorghumHead (https://github.com/oceam/DeepSorghumHead).

### 3.2. Reducing Human Labeling Cost via a Weak Supervision Strategy

**Figure 2:**
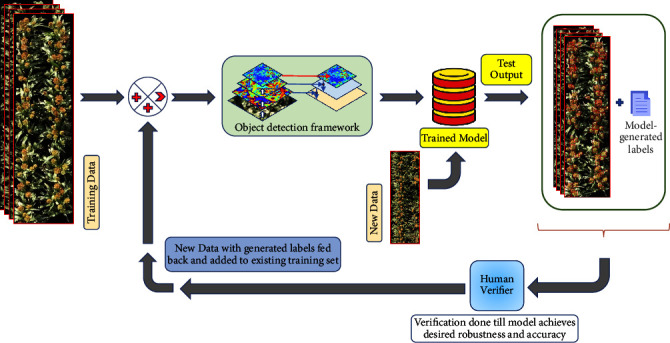
Automated annotation protocol: the initial input image passed through the network is part of the training dataset. This trains the RetinaNet architecture and generates a model, which then is used to annotate new data. This annotation is then verified by human expert raters and fed back into the network as new training data. Process is repeated until network reaches desired level of accuracy.

Here we describe our proposed weak supervision protocol as illustrated in Figure 2. A brief overview of deploying and inner workings of our framework for carrying out the weakly supervised training can be stated as follows: we first start with an initial model trained with a single image (randomly chosen from the pool of 283 images). Upon successful training (all possible hyperparameters optimized as much as possible), we extract a trained model at epoch 40 of the training step. Considering we have only one image, our initial training step is quite fast with each epoch taking about 200*s*. Following our protocol illustrated in Figure 2, we then utilize this semitrained model to generate the labels for another randomly selected image from our 1440-image dataset. The generated labels and the image itself are then fed into the image annotator app for validating the bounding box locations and corrections are made by a human annotator if and as necessary. The corrected labels are then extracted as new labels for the image and we then add this labeled image to our training set. Our training dataset (now consisting of two labeled images) is then used to retrain the deep learning model to build an improved version that performs better than our initial model. We iterate this process over our pool of training samples until we reach our desired performance as determined by evaluating the trained model at the end of every iteration. At the end of this process, we obtain our final model which then can be deployed in real time to predict crop (sorghum)-yield and estimate productivity.

As discussed in the introduction, our primary motivation for introducing this kind of strategy is* active learning*. However, we stop short of calling our framework that and simply refer to it as a* weak supervision* strategy instead, due to the rationale presented below. In the current context, the goal of an active learning framework would be to leverage the semitrained learning model (trained with a small subset of labeled data) to optimally select a subset of (originally) unlabeled training data samples for human labeling/verification. However, in this work, we just* randomly* select such a subset and not use any* optimal* selection procedure. The primary objective here is to show a proof-of-concept that such synthetic annotation can dramatically reduce the cost of human labeling. In our case, we see, approximately, a fourfold reduction in annotation time when a human just verifies/modifies synthetically annotated data by a semitrained model (see Figure 5). However, it is to be noted that framing this problem rigorously as an active learning problem remains a key future research focus for us.

### 3.3. Evaluation Metrics

We use Pearson's linear correlation coefficient, the coefficient of determination (*R*^2^), and mAP (mean average precision) based on an IoU (intersection-over-union) threshold. We also calculate and compare heritability (*h*^2^). These metrics, though well defined in literature, are briefly discussed here for the sake of completeness.

#### 3.3.1. Pearson's Linear Correlation Coefficient (*ρ*) and Coefficient of Determination (*R*^2^)

Given a pair of random variables (*X*, *Y*), Pearson's linear correlation coefficient is defined as

(5) $$\begin{array}{c} \rho_{X,Y}=\frac{\mathrm{cov}\left(X,Y\right)}{\sigma_{X}\sigma_{Y}} \end{array}$$

where cov⁡(*X*, *Y*) denotes the covariance between *X* and *Y* and *σ*_*X*_ and *σ*_*Y*_ denote the standard deviation of *X* and *Y*, respectively. This can be extended to paired data, *X* : (*X*_1_, *X*_2_, …, *X*_*n*_) and *Y* : (*Y*_1_, *Y*_2_, …, *Y*_*n*_), consisting of *n* pairs. In that case, we have

(6) $$\begin{array}{c} \rho =\frac{\sum_{i=1}^{n}\left(x_{i}-\bar{x}\right)\left(y_{i}-\bar{y}\right)}{\sqrt{\sum_{i=1}^{n}{\left(x_{i}-\bar{x}\right)}^{2}}\sqrt{\sum_{i=1}^{n}{\left(x_{i}-\bar{x}\right)}^{2}}} \end{array}$$

where *n* is the sample size, *x*_*i*_, *y*_*i*_ are the individual sample points indexed by *i*, and $\bar{x}=\left(1/n\right)\sum_{i=1}^{n}x_{i}$ and $\bar{y}=\left(1/n\right)\sum_{i=1}^{n}y_{i}$ are the sample means for *X* and *Y*, respectively. The correlation coefficient, *ρ*, can range from -1 to +1. A value of +1 implies that there exists a perfect positive linear correlation between *X* and *Y*; i.e., as *X* increases, so does *Y* following the perfectly positive correlation for all existing points. A value of -1 signifies perfect negative linear correlation; i.e., *X* increases and *Y* decreases following the same perfectly negative correlation for all existing points. A zero correlation indicates there is no linear correlation between *X* and *Y*. For additional information on *ρ*, refer to [45].

Now, the coefficient of determination, *R*^2^, is simply the square of the linear correlation coefficient; i.e., *R*^2^ = *ρ*^2^. *R*^2^ thus takes values between 0 and 1. For additional information on *R*^2^, refer to [45].

#### 3.3.2. Mean Average Precision (mAP)

To provide a brief overview of the mAP measure, we first review a few basic metrics such as True Positive (TP), True Negative (TN), False Positive (FP), and False Negative (FN) measures.

Consider a binary (0, negative, or 1, positive) classification problem. Results that fall under TP are those samples that are truly 1 and have been correctly classified by the machine learning (ML)/ deep learning (DL) model as 1. Results that fall under TN are those samples that are truly 0 and have been correctly classified by the ML/DL model as 0. Results that fall under FP are those samples that are truly 0 and have been incorrectly classified by the ML/DL model as 1 and results that fall under FN are those samples that are truly 1 and have been incorrectly classified by the ML/DL model as 0.

Now, we define two more measures, Precision and Recall. Precision is defined as

(7) $$\begin{array}{c} Precision=\frac{TP}{TP+FP} \end{array}$$

while Recall is defined as

(8) $$\begin{array}{c} Recall=\frac{TP}{TP+FN} \end{array}$$

Upon defining these two measures, we now define average precision (AP). For a ranked sequence of outputs, precision varies with recall and a Precision-Recall curve can be plotted. For precision, *p*(*r*), as a function of recall *r*, the average precision is defined as the area under the P-R curve and is given by

(9) $$\begin{array}{c} AP=\overset{1}{\underset{0}{\int }}p\left(r\right)dr \end{array}$$

For the discrete cases (which is most practical cases) we have

(10) $$\begin{array}{c} AP=\overset{1}{\underset{j=1}{\sum }}P\left(j\right)\Delta r\left(j\right) \end{array}$$

where *j* is the rank in the sequence of samples, *n* is the total number of samples, and *P*(*j*) is the precision at the cut-off point, *k*. Δ*r*(*j*) is change in recall from *j* − 1 to *j*. Once we define average precision (AP), *mAP* for a set of queries is simply defined as the mean of the APs over those queries. Thus, for *q* queries, average precision as a function of those queries, *AP*(*q*), and *Q* being the total number of queries, we have

(11) $$\begin{array}{c} mAP=\frac{1}{Q}\overset{Q}{\underset{q=1}{\sum }}AP\left(q\right) \end{array}$$

For additional information on *mAP*, refer to [46].

#### 3.3.3. Intersection-over-Union (IoU)

**Figure 3:**
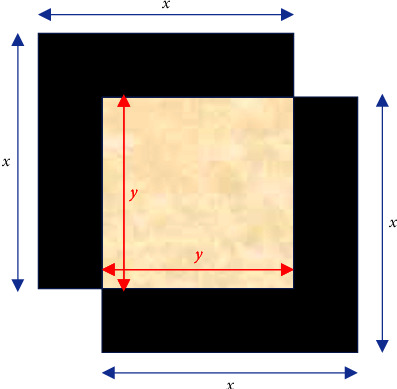
Pictorial representation of IoU.

Also termed Jaccard Index, we define this metric as shown in Figure 3 and the following equation:

(12) $$\begin{array}{c} IoU=\frac{A_{o}}{A_{u}}=\frac{y^{2}}{2x^{2}-y^{2}} \end{array}$$

where *A*_*o*_ is the area of overlap and *A*_*u*_ is the area of union of the two sets. In Figure 3, we represent the sets as squares for the sake of simplicity. For our problem, we report our results considering an IoU threshold of 0.5. We also explore other IoU values ranging from 0.4 to 0.9 and report corresponding mAP values in the discussion section.

For additional information on *IoU*, refer to this source [47].

#### 3.3.4. Heritability of Breeding Population

Plant breeders use the term heritability (or repeatability) as a measure of how much of the variance in a trait (such as head number per plant) is associated with genetic rather than random environmental factors [48]. This statistic can be estimated whenever sets of genotypes are compared (with some degree of environmental replication). If a trait can be measured with high heritability, it means that the breeder can have greater confidence that selecting for the trait is associated with a true genetic rather than environmental or experimental effect. Hence, heritability is a useful statistic to compute in such populations, in addition to standard statistics related to correlation, precision, and recall. For example, heritability was used to evaluate the performance consistency of methods for estimation of plant height [49]. It indicates the proportion of total variation of a trait that is due to differences between genotypic individuals [50]. In the early generation of breeding population, simple traits with high heritability are selected to rapidly fix the associated genes and genomic regions [51]. In this paper, we calculate and compare the heritability of head number from human field counting and the proposed method as well. In the experiment we have done here, the breeding population is at an early generation (still quite diverse and large) and so the estimates of heritability are pertinent to decisions for the plant breeder on whether to use head number or not as a selection trait. Until the development of image-analysis methods, it has been labor-intensive to measure head number, so there is little data available on its heritability.

Formally, we define heritability using a linear mixed model that was designed to estimate the variation components via the restricted log likelihood (REML) method with the R package SpATS [52]. The spatial trend is counted by a two dimensional P-spline (i.e., row and column in the experimental design). Genotype and replicate effects were considered as random factors. With this setup, heritability is calculated as

(13) $$\begin{array}{c} h^{2}=\frac{V_{g}}{V_{t}} \end{array}$$

where *h*^2^ is heritability, *V*_*g*_ is the genotypic variation component, and *V*_*t*_ is the total variation component.

## 4. Results

### 4.1. Weakly Supervised Deep Learning Model Training

**Figure 4:**
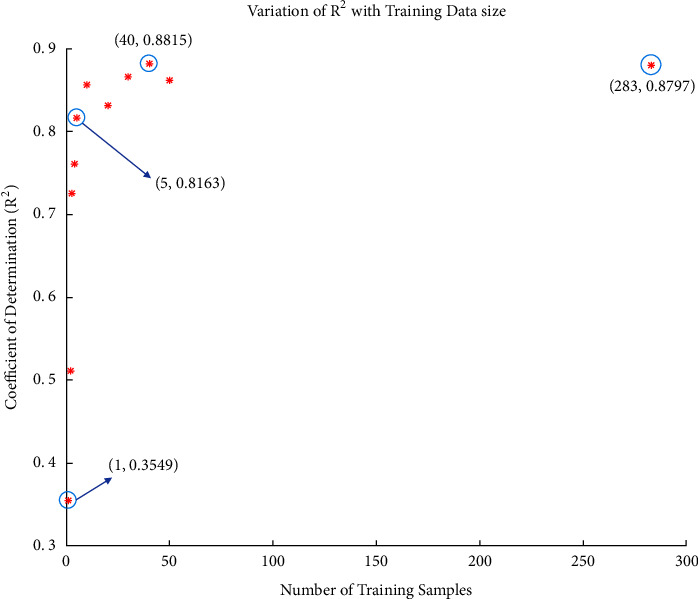
Variation of *R*^2^ for the 1260-test dataset with change in training data size, following our automated annotation protocol (see Figure 2), varying from models trained with 1, 2, 3, 4, 5, 10, 20, 30, 40, 50, and 283 images.

**Figure 5:**
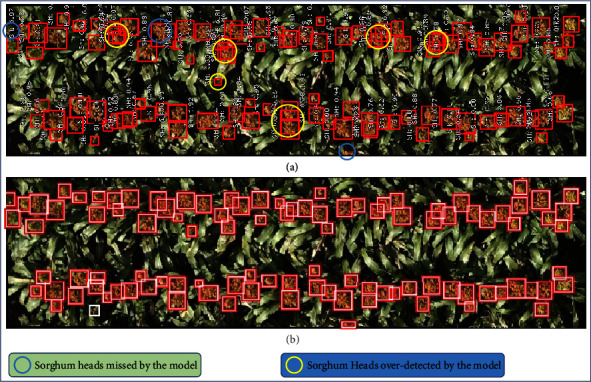
(a) Model output image (about 180 seconds for verifying and making corrections) versus (b) manually annotated image starting from scratch (about 720 seconds to label the entire image).

Following our protocol as described in the first paragraph of Section 3.2 (see Figure 2), we observe that *R*^2^ value for 2-image model is significantly greater than the *R*^2^ value for the first-step single-image model (see Figure 4). We keep iterating until we reach a high *R*^2^ value of 0.8815 for the model trained with 40 randomly selected images, after which the *R*^2^ value decreases slightly for the 50-image model. For the set of all 283 images that we set aside initially as our training set, we see *R*^2^ = 0.8797 (which is still slightly lower than *R*^2^ for the 40-image trained model). In Figure 5, we show the model output for an image tested with the 5-image trained model (Figure 5(a)) and compare it with the manually verified/corrected image (Figure 5(b)). The true label count for the image was 104 sorghum heads which took about 720 seconds to be manually labeled from scratch. On the other hand, using the 5-image model, the corrections that had to be made were only for six overdetected sorghum heads and three undetected heads. Typically, overdetection happens only when there are multiple sorghum heads in close proximity, which drives the model to predict more heads at such locations than the actual number. Missed detection can be attributed to the significantly low number of training samples (five samples only in this case). Making these few corrections took about 180 seconds. In majority of the cases, when the model output images are fed back into the image annotator app, we note about 4 times speed up on an average as compared to the manual annotation process from scratch. Evidently, this depends on how fast the human expert performs the annotations and subsequent validations. The quality of the semitrained model being used to generate the labels in the first place is also a critical factor. Thus, this kind of approach provides a significant advantage in terms of being a lot less expensive and more time-efficient without sacrificing performance.

**Figure 6:**
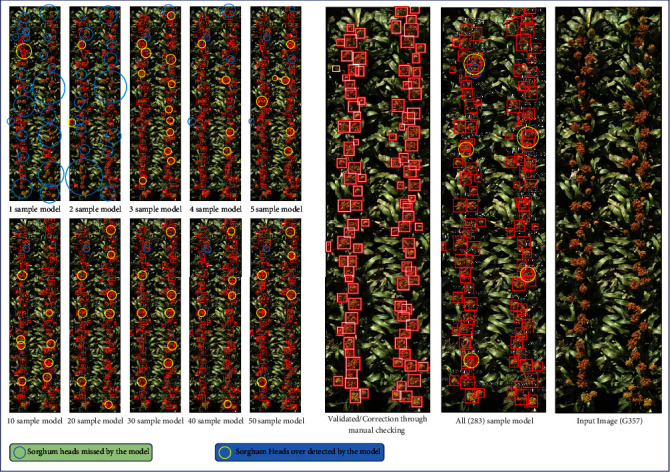
Comparing results for a randomly selected test sample (genotype G357) for different models, starting from model trained with one sample and passed through our weak supervision based learning protocol using 2, 3, 4, 5, 10, 20, 30, 40, 50, and 283 training samples.

Figure 6 shows qualitative comparison between the models trained with varying number of training samples, starting from 1 image to 5 images with increment of 1 and then from 10 images to 50 images with increment of 10 and finally using all the available 283 training images at our disposal. We randomly select an image from our 1260-image test dataset and get the outputs for different models. Clearly we see here that the 1-image model shows the poorest performance (*R*^2^ = 0.3549; see Figure 4) with several sorghum heads missed. As we progress further, we see that the number of missed detections fall down to just 2 for the 5-image trained model (*R*^2^ = 0.8163; see Figure 4) and then finally to 1 for the rest of the models (trained with 10, 20, 30, 40, 50, and 283 images). We observe that the one sorghum head that remains undetected is in extreme close proximity to another head. Hence, accurate counting in this case becomes difficult even for human experts. The other problem that arises when following the automated annotation protocol is overdetection of sorghum heads, which also happens when multiple heads are in close proximity. From Figure 6, we note that overdetections are there even for the model trained with all available training data (283 images), although much less compared to the earlier models (1,2,3 image based models for instance).

### 4.2. Evaluating the Proposed Framework

**Figure 7:**
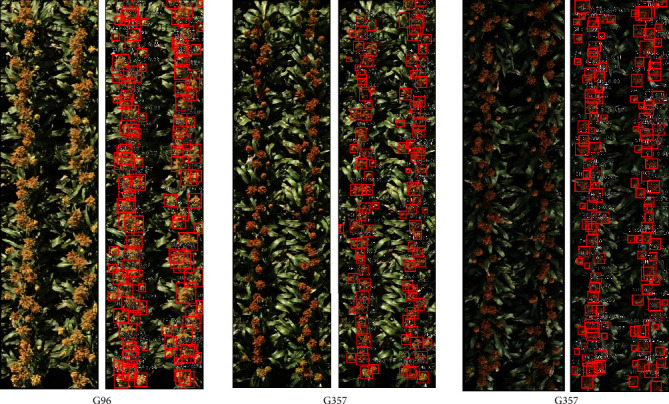
Detection results for Sorghum Plot images across different genotypes, randomly chosen from the 1260-image test dataset. The model is able to detect the sorghum heads irrespective of image-capture conditions (bright, sunny to dark and shadowy), shape/size of heads, color of heads, ranging from light to deep brown as well as orange (due to different genotypes).

**Figure 8:**
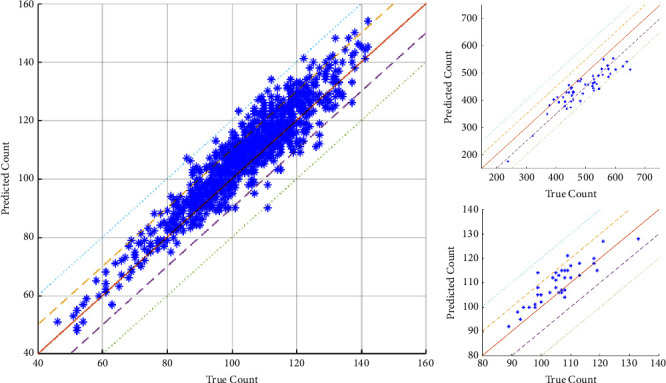
Correlation plots for: Test 1260-image test dataset (left) with *ρ* = 0.9389, *R*^2^ = 0.8815 @ mAP of 0.9413 (IoU threshold = 0.5), test dataset labeled “52Cropped” (right-top) with *ρ* = 0.9063, *R*^2^ = 0.8214, and test dataset labeled “40Plots” (right-bottom) with *ρ* = 0.8756, *R*^2^ = 0.7667.

We initially set out to test our model on our test dataset consisting of 1269 hand-labeled images. However, out of those 1269 images, nine images have white sorghum heads that were not represented at all in the training set. Hence, we do not consider those images for calculating *ρ* and *R*^2^ values and report metrics based on only 1260 test images.  Figure 7 and Figure S2 show the detection results. The detected sorghum heads are shown within red bounding boxes. It is observed that the model effectively detects all the sorghum heads across different genotypes: G96 and G357 (captured in bright and dark conditions). A few more examples are provided in the Supplementary Information. The coefficient of determination (*R*^2^) between the manual and proposed method of counting achieved is 0.8815. This implies that the model performs quite well in estimating the sorghum head counts, which, in turn, leads to getting accurate estimates of overall crop yield. The manual counting is a two-pronged approach; first, from the RGB images, a manual labeling of all distinctly visible heads was done such that the number of labeled bounding boxes is same as the manual count from the image. The second method involves a field count where, in the field, for each plot, breeders put a 1-meter stick randomly inside a plot and counted the number of sorghum heads around it. The manual count from the RGB images are used to calculate the correlation plot, shown in Figure 8. On the other hand, the manual count from the field is used to calculate heritability, as reported in the next paragraph. We, however, do not consider calculating the count correlation between the field count and that based our proposed approach. Note that we also use two separate datasets (“52Croppped” and “40Plots”) from our previous work [24] in Figure 8.

The heritabilities were 0.31 and 0.65 for head numbers by human field counting (counted heads in a 1 linear meter per plot) and by the proposed method (counted heads for entire plot), respectively. A higher heritability indicates that the proposed method is able to capture the genotypic variations in the field from where the data was collected. This higher heritability indicates that the proposed method is more reliable, and the counted head number could be used as a selection trait for breeding experiments.

## 5. Discussion

While our approach is based on the established principles of deep learning for object detection and localization, it is quite different from traditional object detection tasks carried out for datasets such as ImageNet [53] and COCO [54] in the following ways.

- First of all, there is a limited availability of publicly available annotated agricultural data which reduces the possibility of obtaining high-performance object detection/localization models through transfer learning.
- Many agricultural image data such as the one considered here demonstrate a higher level of occlusion and background clutter (leading to higher possibilities of confusing objects of interest with background elements) compared to other traditional image datasets.
- Environmental variations (e.g., cloudy sky, windy weather) impact the agricultural images in a significant way and hence, make them harder to work with. Similarly, data samples are also sensitive to imaging angles, field conditions and plant genotypes. Hence, the robustness requirements are significantly high for the learning models built for agricultural applications.

Considering all the complexities in dealing with our data (as stated above), our model is observed to be robust and at the same time sufficiently accurate in detecting the sorghum heads leading to a good estimate of the crop yield. The model also helps in future annotations of new data which can be fed back to the neural network to further improve the model via weak supervision.

A key point to note in Figure 8 is that, for most cases, the model overestimates the sorghum headcounts. For most of these cases, the overestimate is at most by 5 or 10 counts only for the 1260-image and the “40Plots” test set. This overestimation is primarily due to the fact that some of the heads are very close to each other and overlapping. This results in the model mapping more bounding boxes per sorghum head (i.e., more than 1 bounding box for 1 sorghum head) than required for those clusters. This leads to the model detecting more heads than what is actually present in those regions. In case of the “52Cropped” dataset, the model underestimates the count due to the presence of white sorghum heads, of which there were no samples in the training set.

### 5.1. Comparison with State-of-the-Art

**Figure 9:**
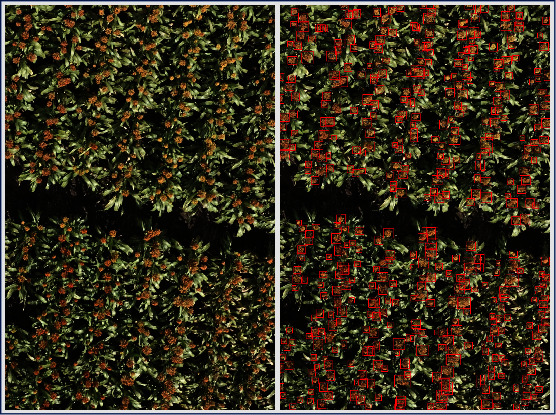
Model output for a sample from the “52Cropped” data.

We notice a significant performance improvement in this study over our previous work [24]. In the previous method [24], the authors test their method on the “52Cropped” and the “40Plots” datasets where they achieve a *R*^2^ value of 0.84 and 0.56, respectively. On the other hand, our method achieves 0.82 and 0.77 *R*^2^ values on the same datasets. Thus our model performs just as well for the “52Cropped” dataset and significantly better for the “40Plots” dataset. However, we note that the “52Cropped” dataset has images with a significant number of white sorghum heads, which were not represented at all in our training dataset. Even then our model was able to detect a good number of these heads and achieve a similar level of performance with the previous best. This leads us to believe that adding white sorghum head data to the already existing training dataset would significantly increase the *R*^2^ values for this particular dataset as well. This is left as a future direction and continuation of this work. An example output for a sample image from the “52Cropped” dataset is shown in Figure 9.

### 5.2. Visualizing Learned Features

**Figure 10:**
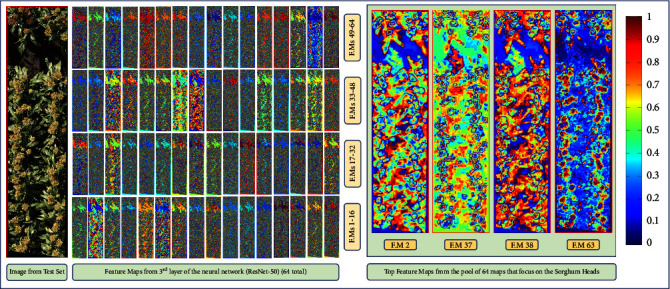
Feature Map Visualization for test image from genotype G40.

We prune target layers of the neural network to see what features are learned hierarchically as the image is passed through during the testing phase.  Figure 10 shows such an example. As we can see, the residual network is able to learn the sorghum head shapes and is able to clearly capture the different head sizes as well. We put forth this visualization as a trust mechanism that gives the user information on whether the neural network actually learns important visual cues comparable to what humans would usually look for when performing manual surveys in the field. It turns out that, though not comparable to a human expert level, deep neural networks do learn orientation as well as the shape and size of the sorghum heads as can be seen from Figure 10. Here we take a random test sample and visualize all the 64 feature maps from the 3^*rd*^ layer of the network and choose the top four among them that actually inspect the sorghum heads. The network is also able to discern overlapping sorghum heads and approximately demarcate boundaries separating one sorghum head from another even in the cases of substantial overlap (as can be seen in feature map 37 and feature map 63 in Figure 10). From a counting perspective, this discerning capability of the model is crucial for getting an accurate count.

Looking at Figure S7, we see that, for the feature maps for the brown sorghum heads, the trained model is able to distinctly bring out the sorghum heads whereas, for the white heads, it fails to discern a proper boundary for each head. Comparing the four feature maps (feature map 2, 37, 38, and 63) for both samples, we observe that the feature maps for the brown heads highlight the peripheral regions for each of the heads within the sample whereas it is not so for the white-head sample.

### 5.3. Conclusion and Future Works

A few conclusions that we draw from our study are summarized as follows:

- Proposed weak supervision strategy significantly increases the efficiency of training data generation, which otherwise can be an extremely tedious, expensive and time-consuming process.
- Very few hand-labeled data samples (40 images) were sufficient to achieve the desirable results.
- Proposed deep learning model is sufficiently robust to varying orientations as well as different lighting conditions.
- A high counting accuracy (*R*^2^ = 0.8815 for plot level) at a high *mAP* of 0.94 with *IoU* ≥ 0.5 was achieved on our 1260-image test set.
- An intuitive but still interesting observation through this work was that for object detection in the agriculture domain, labeling similar image does not increase the accuracy of the model. Here “similar” images refer to two different image samples having the same sorghum genotype instances, i.e., images having sorghum heads of very similar shape, size, and color.

Future investigations will include (but not limited to):

- Evolving the current weak supervision strategy to a fully functional active learning methodology
- Possibilities of real time counting by deploying the trained model on UAVs/ground robots and smart-phones
- Incorporating further diversity (e.g., white sorghum head images) into the training dataset
- Developing a domain knowledge-based strategy for automated and smart selection of “distinct” training images. This stems directly from the final point made in the conclusion section

## Supplementary Materials

Supplementary Materials
* Choice of IoU Threshold*. As seen in Figure S4, we chose an optimum IoU threshold of 0.5, as anything less than that gives us additional bounding boxes for every detected sorghum head and affects the *mAP* values. Changing the IoU threshold, however, does not affect the *R*^2^ value and it remains constant at 0.88 for our 1260-image test set. Thus, for most cases, choosing *IoU* > = 0.5 is a standard practice and we follow this.* Robustness to Varying Orientations.* The model is also robust to varying orientations and augmentations of the plot images. This is a very interesting aspect as in most practical cases (e.g., when deploying the model on drones or on ground robots), it is not always possible to take perfectly vertically oriented images or videos of the plots that match those in our training dataset. Thus, being robust to augmentations even when trained on handpicked images (that represent about 2 vertical crop columns each) is an extremely desirable quality for such a framework (see Figure S5).* Unseen Cases*. The model fails in cases where the sorghum heads are very small (less than 32 area pixels in size) and in cases where a completely different genotype depicting white sorghum heads are present. While the case for small sorghum heads is taken care of by resizing the image to an appropriate size where no sorghum head shape go below the lower limit of 32-area pixel area, the genotype issue can only be addressed by including such examples in the training dataset. This genotype is identified as G286. The model fails to detect these white sorghum heads in the first place, as there were no such training examples in the training dataset to start with. Figure S6 shows such an example. We also visualize the learned features for two such white-head samples to see what the network looks at for these cases. This is shown in Figure S7 (right). A point to note here is that, even though the network had no white-head training samples to begin with, it can capture the shape-size features for some of these heads, but then fails for others. Figure S1: web-based annotation tool used for labeling data. Here, the left panel shows labeling site, where labeling is done from scratch and the right panel shows validation site, where we validate the prelabeled data (prelabeled either by human annotators or the deep learning model). Figure S2: more detection results for Sorghum Plot images across different genotypes and variations. Figure S3: variation of *R*^2^ for the 1260-test dataset with change in orientation. The *R*^2^ values are for the trained model (trained with 40 images). Figure S4: the variation of mAP with respect to IoU shows that mAP begins to drop drastically after the 0.6 IoU threshold. Thus, for simplicity and getting satisfactory outputs from the model, an IoU threshold of 0.5 is chosen, which is a widely accepted threshold in the deep learning and object detection/localization community. Figure S5: model output for varying orientations. Here we take the original image from the test set and perform several counterclockwise (CCW) rotations and then test our model on these images. As can be seen from the detection results, the model accurately picks up on all the sorghum heads in the original as well as the transformed images. Figure S6: failure case: Genotype G286 (white heads). Although the model had no white-head samples to train on, it is still able to detect some as seen in the sample to the right. Figure S7: Feature Map Visualization for brown sorghum head (left) and white sorghum head (right). For the brown sorghum head, we see that framework specifically picks up the region around the heads while it does not do the same for the white heads.
