# Supplementary figures and images for: A Weakly Supervised Deep Learning Framework for Sorghum Head Detection and Counting
Source: Plant Phenomics. 2019 Jun 27;2019:1525874. doi: 10.34133/2019/1525874 (PMC7706102; doi:10.34133/2019/1525874)

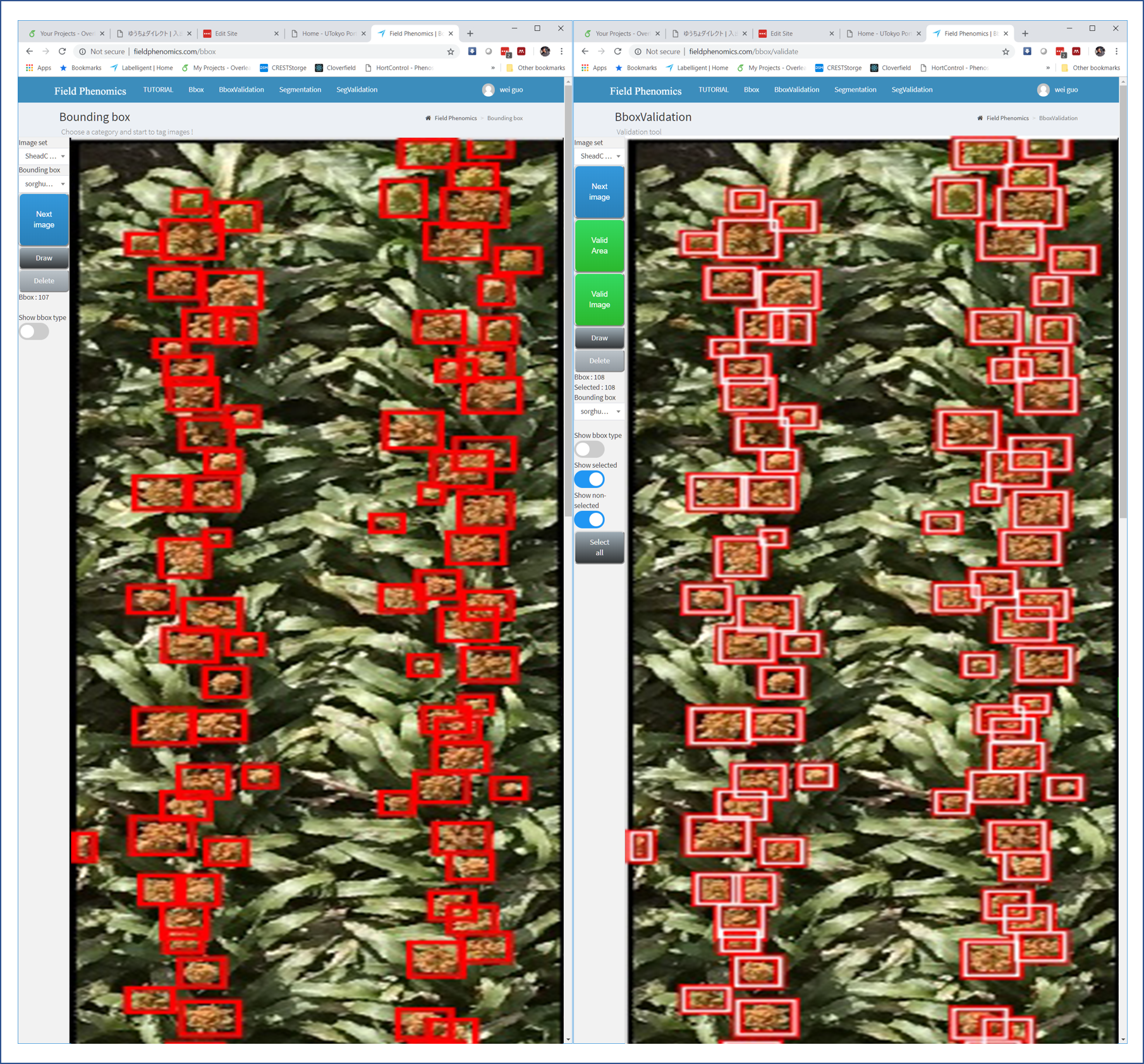

Supplement: Supplementary Materials — Choice of IoU Threshold. As seen in Figure S4, we chose an optimum IoU threshold of 0.5, as anything less than that gives us additional bounding boxes for every detected sorghum head and affects the mAP values. Changing the IoU threshold, however, does not affect the R2 value and it remains constant at 0.88 for our 1260-image test set. Thus, for most cases, choosing IoU > = 0.5 is a standard practice and we follow this. Robustness to Varying Orientations. The model is also robust to varying orientations and augmentations of the plot images. This is a very interesting aspect as in most practical cases (e.g., when deploying the model on drones or on ground robots), it is not always possible to take perfectly vertically oriented images or videos of the plots that match those in our training dataset. Thus, being robust to augmentations even when trained on handpicked images (that represent about 2 vertical crop columns each) is an extremely desirable quality for such a framework (see Figure S5). Unseen Cases. The model fails in cases where the sorghum heads are very small (less than 32 area pixels in size) and in cases where a completely different genotype depicting white sorghum heads are present. While the case for small sorghum heads is taken care of by resizing the image to an appropriate size where no sorghum head shape go below the lower limit of 32-area pixel area, the genotype issue can only be addressed by including such examples in the training dataset. This genotype is identified as G286. The model fails to detect these white sorghum heads in the first place, as there were no such training examples in the training dataset to start with. Figure S6 shows such an example. We also visualize the learned features for two such white-head samples to see what the network looks at for these cases. This is shown in Figure S7 (right). A point to note here is that, even though the network had no white-head training samples to begin with, it can capture the shape-size feat [file 1525874.f1.zip › 1525874.f1/figS1_web_tool1.png]

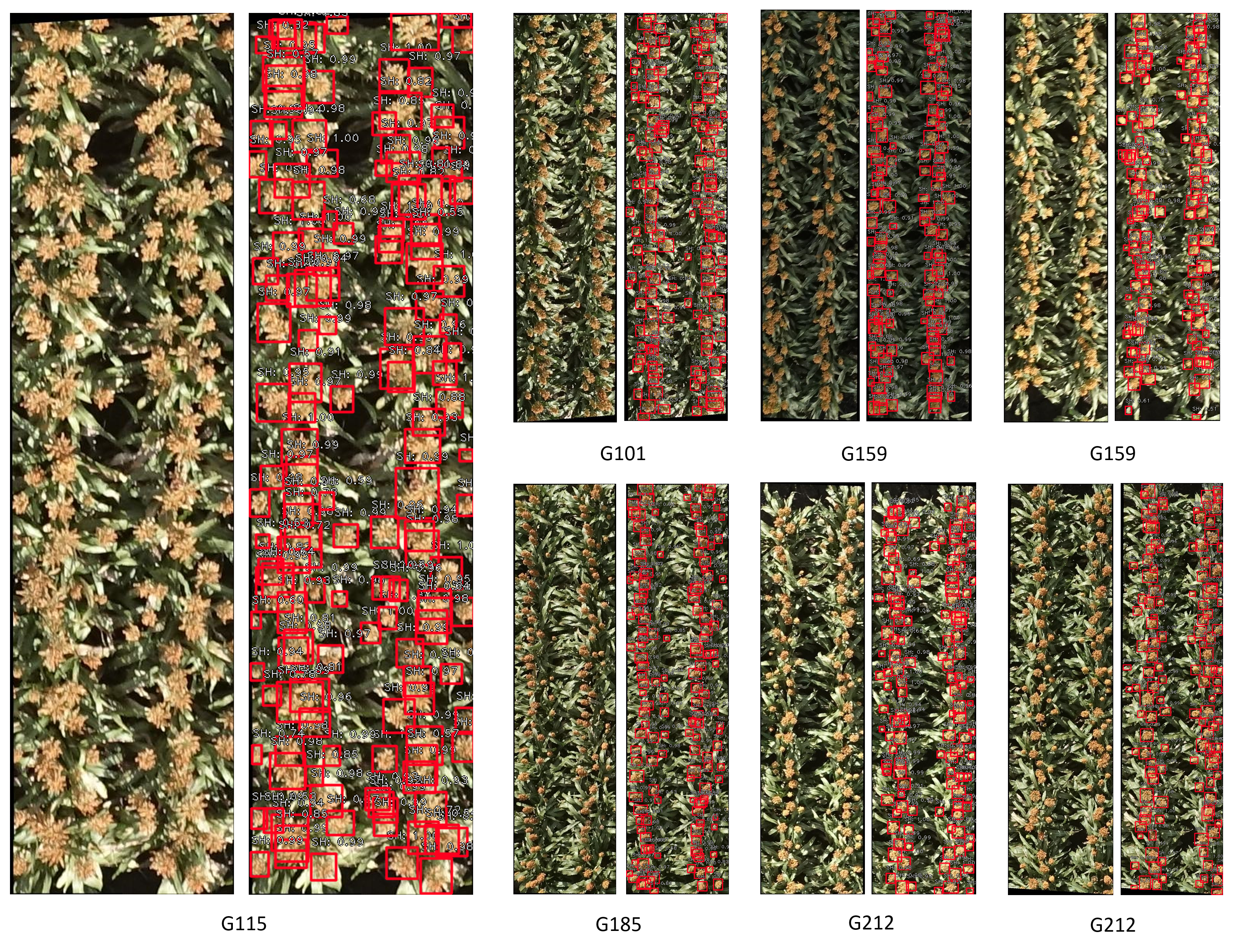

Supplement: Supplementary Materials — Choice of IoU Threshold. As seen in Figure S4, we chose an optimum IoU threshold of 0.5, as anything less than that gives us additional bounding boxes for every detected sorghum head and affects the mAP values. Changing the IoU threshold, however, does not affect the R2 value and it remains constant at 0.88 for our 1260-image test set. Thus, for most cases, choosing IoU > = 0.5 is a standard practice and we follow this. Robustness to Varying Orientations. The model is also robust to varying orientations and augmentations of the plot images. This is a very interesting aspect as in most practical cases (e.g., when deploying the model on drones or on ground robots), it is not always possible to take perfectly vertically oriented images or videos of the plots that match those in our training dataset. Thus, being robust to augmentations even when trained on handpicked images (that represent about 2 vertical crop columns each) is an extremely desirable quality for such a framework (see Figure S5). Unseen Cases. The model fails in cases where the sorghum heads are very small (less than 32 area pixels in size) and in cases where a completely different genotype depicting white sorghum heads are present. While the case for small sorghum heads is taken care of by resizing the image to an appropriate size where no sorghum head shape go below the lower limit of 32-area pixel area, the genotype issue can only be addressed by including such examples in the training dataset. This genotype is identified as G286. The model fails to detect these white sorghum heads in the first place, as there were no such training examples in the training dataset to start with. Figure S6 shows such an example. We also visualize the learned features for two such white-head samples to see what the network looks at for these cases. This is shown in Figure S7 (right). A point to note here is that, even though the network had no white-head training samples to begin with, it can capture the shape-size feat [file 1525874.f1.zip › 1525874.f1/figS2_Re_out2.png]

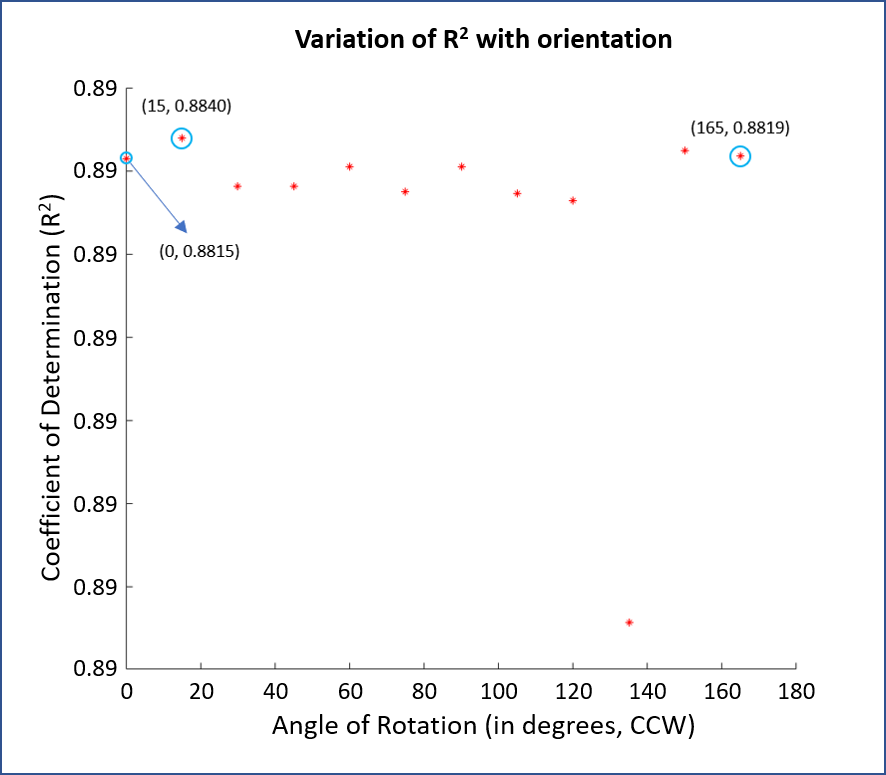

Supplement: Supplementary Materials — Choice of IoU Threshold. As seen in Figure S4, we chose an optimum IoU threshold of 0.5, as anything less than that gives us additional bounding boxes for every detected sorghum head and affects the mAP values. Changing the IoU threshold, however, does not affect the R2 value and it remains constant at 0.88 for our 1260-image test set. Thus, for most cases, choosing IoU > = 0.5 is a standard practice and we follow this. Robustness to Varying Orientations. The model is also robust to varying orientations and augmentations of the plot images. This is a very interesting aspect as in most practical cases (e.g., when deploying the model on drones or on ground robots), it is not always possible to take perfectly vertically oriented images or videos of the plots that match those in our training dataset. Thus, being robust to augmentations even when trained on handpicked images (that represent about 2 vertical crop columns each) is an extremely desirable quality for such a framework (see Figure S5). Unseen Cases. The model fails in cases where the sorghum heads are very small (less than 32 area pixels in size) and in cases where a completely different genotype depicting white sorghum heads are present. While the case for small sorghum heads is taken care of by resizing the image to an appropriate size where no sorghum head shape go below the lower limit of 32-area pixel area, the genotype issue can only be addressed by including such examples in the training dataset. This genotype is identified as G286. The model fails to detect these white sorghum heads in the first place, as there were no such training examples in the training dataset to start with. Figure S6 shows such an example. We also visualize the learned features for two such white-head samples to see what the network looks at for these cases. This is shown in Figure S7 (right). A point to note here is that, even though the network had no white-head training samples to begin with, it can capture the shape-size feat [file 1525874.f1.zip › 1525874.f1/figS3_R2_var_orient_F.png]

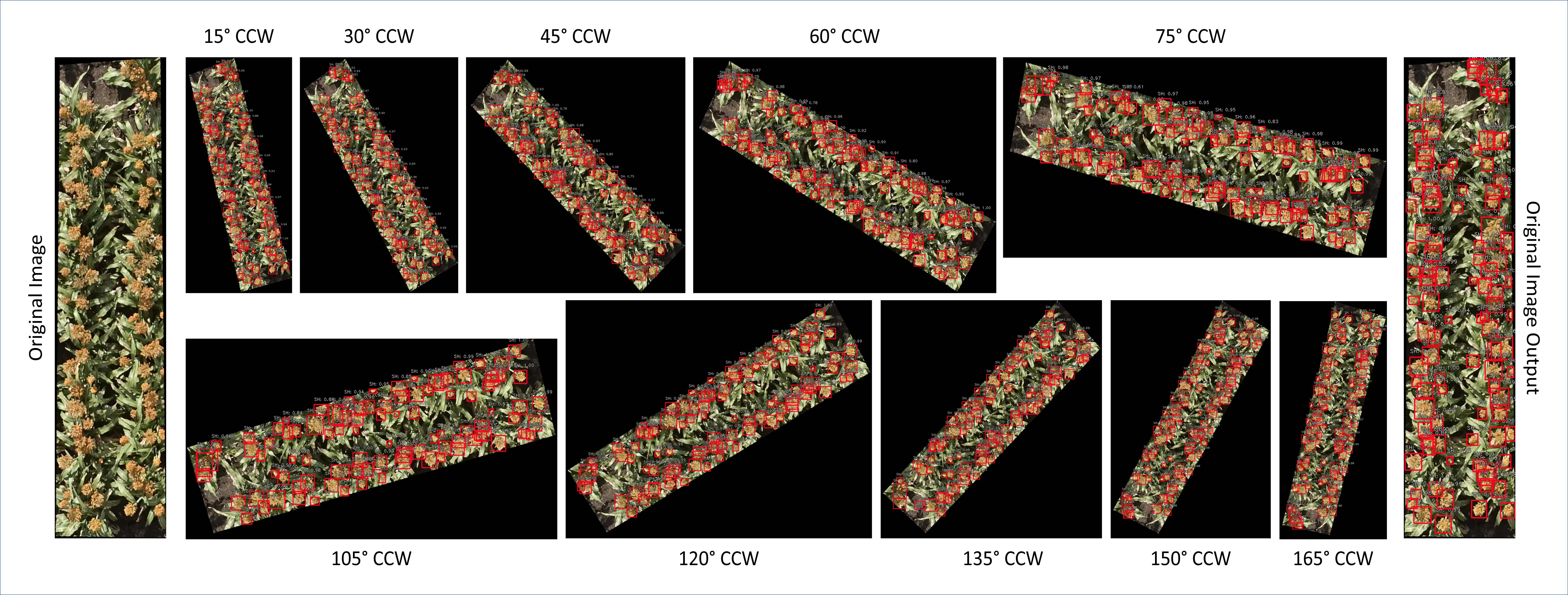

Supplement: Supplementary Materials — Choice of IoU Threshold. As seen in Figure S4, we chose an optimum IoU threshold of 0.5, as anything less than that gives us additional bounding boxes for every detected sorghum head and affects the mAP values. Changing the IoU threshold, however, does not affect the R2 value and it remains constant at 0.88 for our 1260-image test set. Thus, for most cases, choosing IoU > = 0.5 is a standard practice and we follow this. Robustness to Varying Orientations. The model is also robust to varying orientations and augmentations of the plot images. This is a very interesting aspect as in most practical cases (e.g., when deploying the model on drones or on ground robots), it is not always possible to take perfectly vertically oriented images or videos of the plots that match those in our training dataset. Thus, being robust to augmentations even when trained on handpicked images (that represent about 2 vertical crop columns each) is an extremely desirable quality for such a framework (see Figure S5). Unseen Cases. The model fails in cases where the sorghum heads are very small (less than 32 area pixels in size) and in cases where a completely different genotype depicting white sorghum heads are present. While the case for small sorghum heads is taken care of by resizing the image to an appropriate size where no sorghum head shape go below the lower limit of 32-area pixel area, the genotype issue can only be addressed by including such examples in the training dataset. This genotype is identified as G286. The model fails to detect these white sorghum heads in the first place, as there were no such training examples in the training dataset to start with. Figure S6 shows such an example. We also visualize the learned features for two such white-head samples to see what the network looks at for these cases. This is shown in Figure S7 (right). A point to note here is that, even though the network had no white-head training samples to begin with, it can capture the shape-size feat [file 1525874.f1.zip › 1525874.f1/figS4_rotations.png]

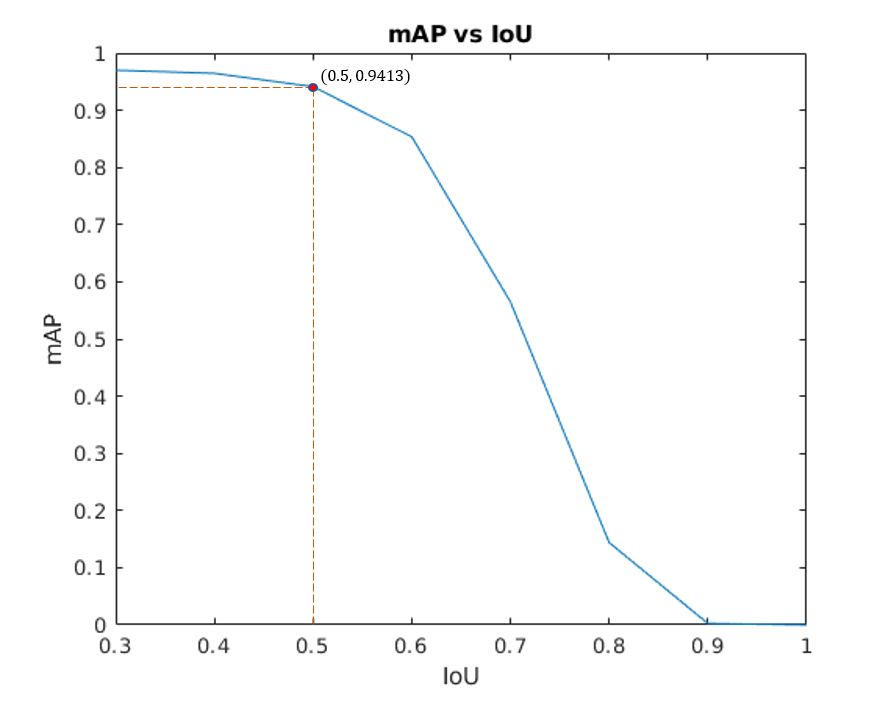

Supplement: Supplementary Materials — Choice of IoU Threshold. As seen in Figure S4, we chose an optimum IoU threshold of 0.5, as anything less than that gives us additional bounding boxes for every detected sorghum head and affects the mAP values. Changing the IoU threshold, however, does not affect the R2 value and it remains constant at 0.88 for our 1260-image test set. Thus, for most cases, choosing IoU > = 0.5 is a standard practice and we follow this. Robustness to Varying Orientations. The model is also robust to varying orientations and augmentations of the plot images. This is a very interesting aspect as in most practical cases (e.g., when deploying the model on drones or on ground robots), it is not always possible to take perfectly vertically oriented images or videos of the plots that match those in our training dataset. Thus, being robust to augmentations even when trained on handpicked images (that represent about 2 vertical crop columns each) is an extremely desirable quality for such a framework (see Figure S5). Unseen Cases. The model fails in cases where the sorghum heads are very small (less than 32 area pixels in size) and in cases where a completely different genotype depicting white sorghum heads are present. While the case for small sorghum heads is taken care of by resizing the image to an appropriate size where no sorghum head shape go below the lower limit of 32-area pixel area, the genotype issue can only be addressed by including such examples in the training dataset. This genotype is identified as G286. The model fails to detect these white sorghum heads in the first place, as there were no such training examples in the training dataset to start with. Figure S6 shows such an example. We also visualize the learned features for two such white-head samples to see what the network looks at for these cases. This is shown in Figure S7 (right). A point to note here is that, even though the network had no white-head training samples to begin with, it can capture the shape-size feat [file 1525874.f1.zip › 1525874.f1/figS5_mAP_IoU.png]

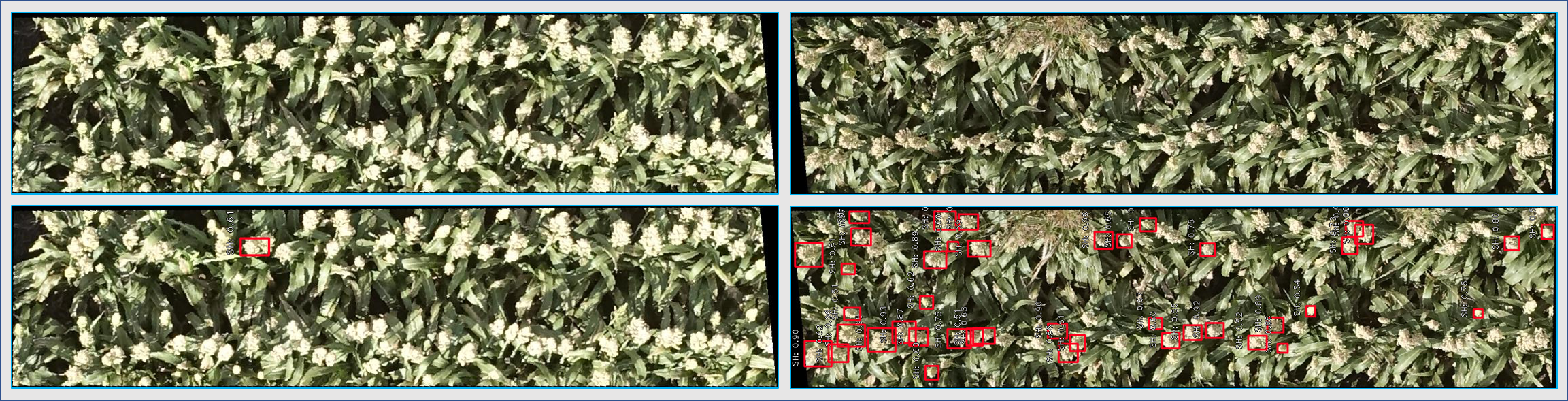

Supplement: Supplementary Materials — Choice of IoU Threshold. As seen in Figure S4, we chose an optimum IoU threshold of 0.5, as anything less than that gives us additional bounding boxes for every detected sorghum head and affects the mAP values. Changing the IoU threshold, however, does not affect the R2 value and it remains constant at 0.88 for our 1260-image test set. Thus, for most cases, choosing IoU > = 0.5 is a standard practice and we follow this. Robustness to Varying Orientations. The model is also robust to varying orientations and augmentations of the plot images. This is a very interesting aspect as in most practical cases (e.g., when deploying the model on drones or on ground robots), it is not always possible to take perfectly vertically oriented images or videos of the plots that match those in our training dataset. Thus, being robust to augmentations even when trained on handpicked images (that represent about 2 vertical crop columns each) is an extremely desirable quality for such a framework (see Figure S5). Unseen Cases. The model fails in cases where the sorghum heads are very small (less than 32 area pixels in size) and in cases where a completely different genotype depicting white sorghum heads are present. While the case for small sorghum heads is taken care of by resizing the image to an appropriate size where no sorghum head shape go below the lower limit of 32-area pixel area, the genotype issue can only be addressed by including such examples in the training dataset. This genotype is identified as G286. The model fails to detect these white sorghum heads in the first place, as there were no such training examples in the training dataset to start with. Figure S6 shows such an example. We also visualize the learned features for two such white-head samples to see what the network looks at for these cases. This is shown in Figure S7 (right). A point to note here is that, even though the network had no white-head training samples to begin with, it can capture the shape-size feat [file 1525874.f1.zip › 1525874.f1/figS6_G286.png]

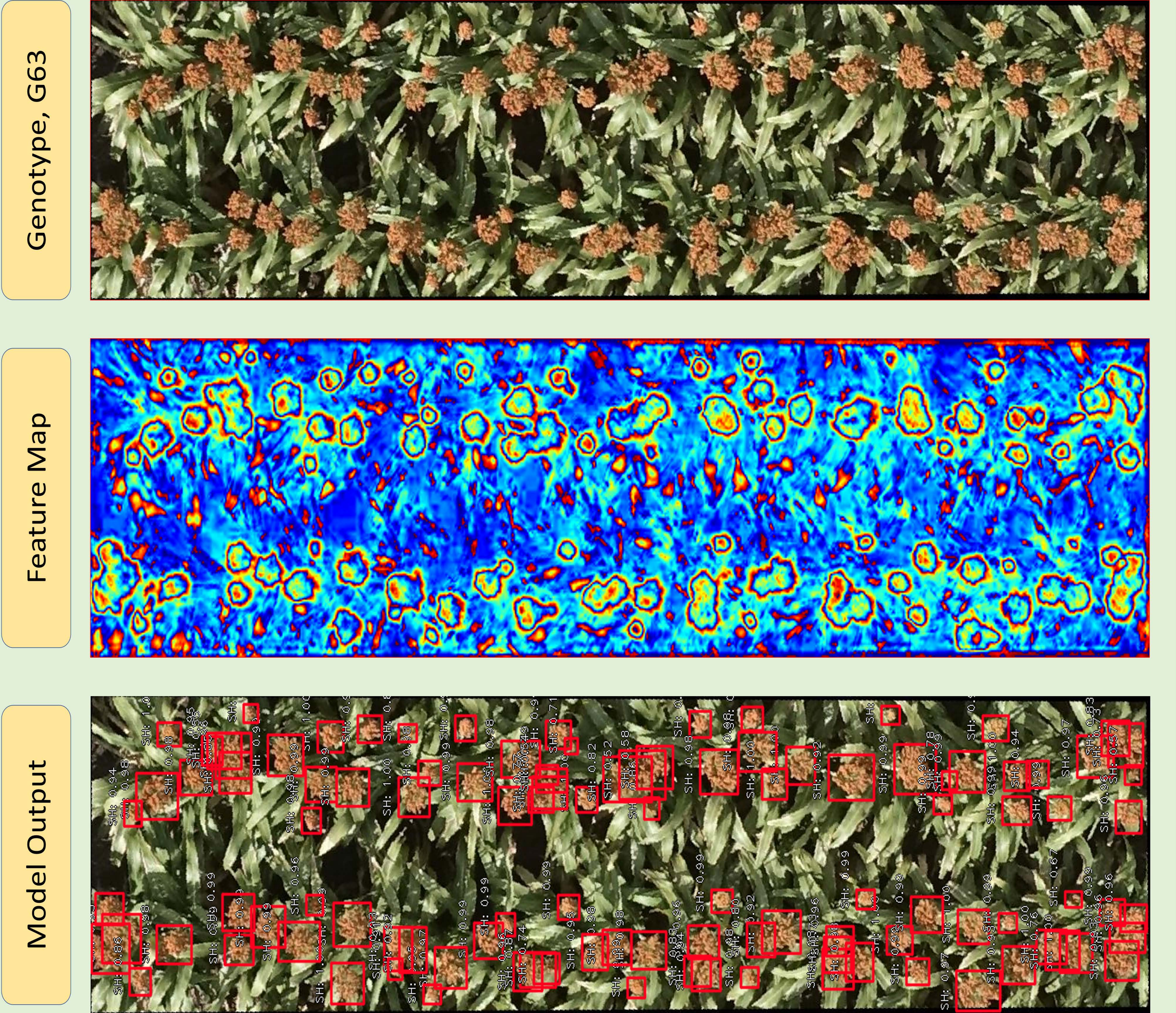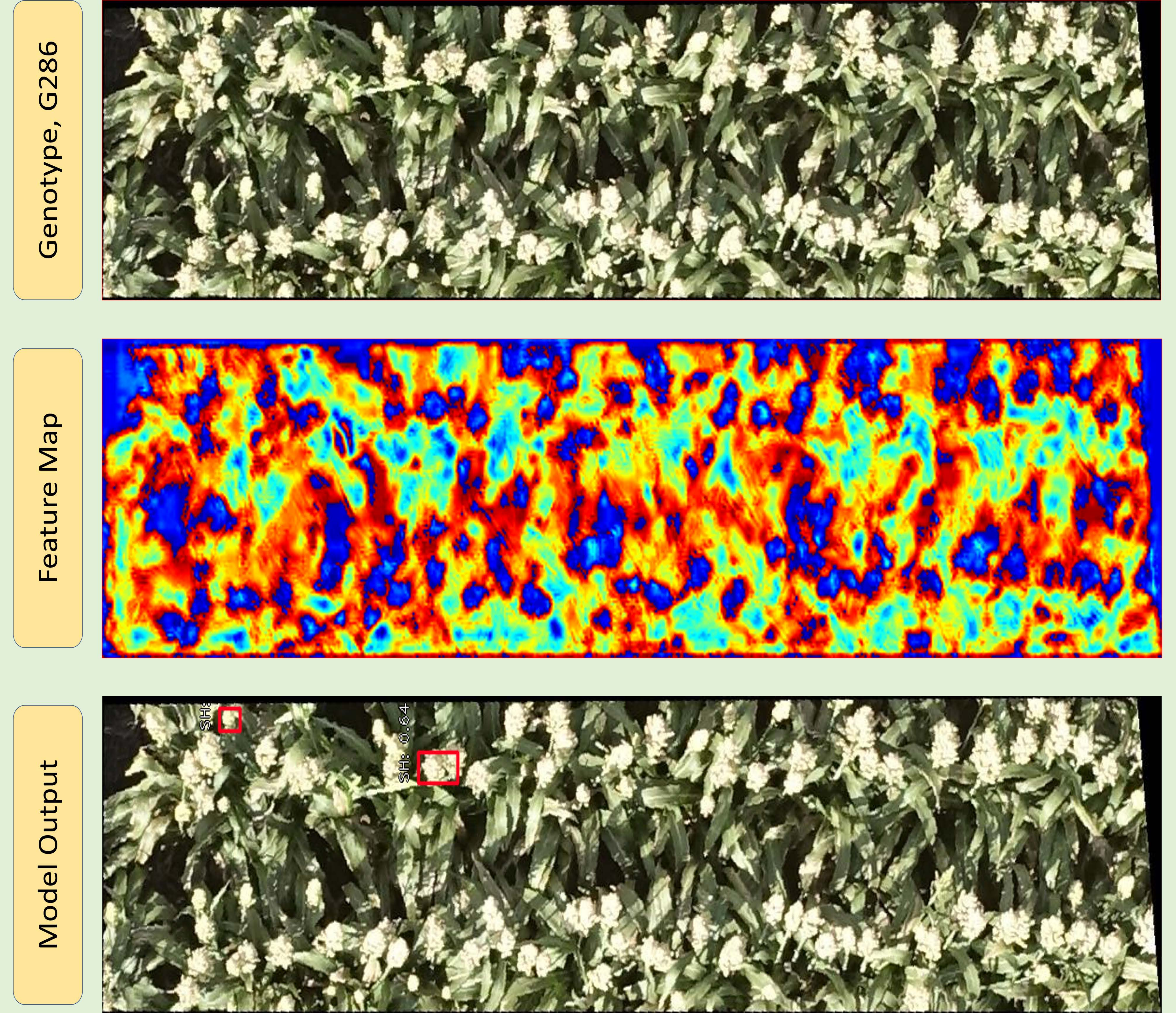

Supplement: Supplementary Materials — Choice of IoU Threshold. As seen in Figure S4, we chose an optimum IoU threshold of 0.5, as anything less than that gives us additional bounding boxes for every detected sorghum head and affects the mAP values. Changing the IoU threshold, however, does not affect the R2 value and it remains constant at 0.88 for our 1260-image test set. Thus, for most cases, choosing IoU > = 0.5 is a standard practice and we follow this. Robustness to Varying Orientations. The model is also robust to varying orientations and augmentations of the plot images. This is a very interesting aspect as in most practical cases (e.g., when deploying the model on drones or on ground robots), it is not always possible to take perfectly vertically oriented images or videos of the plots that match those in our training dataset. Thus, being robust to augmentations even when trained on handpicked images (that represent about 2 vertical crop columns each) is an extremely desirable quality for such a framework (see Figure S5). Unseen Cases. The model fails in cases where the sorghum heads are very small (less than 32 area pixels in size) and in cases where a completely different genotype depicting white sorghum heads are present. While the case for small sorghum heads is taken care of by resizing the image to an appropriate size where no sorghum head shape go below the lower limit of 32-area pixel area, the genotype issue can only be addressed by including such examples in the training dataset. This genotype is identified as G286. The model fails to detect these white sorghum heads in the first place, as there were no such training examples in the training dataset to start with. Figure S6 shows such an example. We also visualize the learned features for two such white-head samples to see what the network looks at for these cases. This is shown in Figure S7 (right). A point to note here is that, even though the network had no white-head training samples to begin with, it can capture the shape-size feat [file 1525874.f1.zip › 1525874.f1/figS7_FM_vis2a_2.pdf]
